# Composite cell sheet for periodontal regeneration: crosstalk between different types of MSCs in cell sheet facilitates complex periodontal-like tissue regeneration

**DOI:** 10.1186/s13287-016-0417-x

**Published:** 2016-11-14

**Authors:** Hao Zhang, Shiyu Liu, Bin Zhu, Qiu Xu, Yin Ding, Yan Jin

**Affiliations:** 1 State Key Laboratory of Military Stomatology, National Clinical Research Center for Oral Diseases, Center for Tissue Engineering, School of Stomatology, The Fourth Military Medical University, Xi’an, Shaanxi 710032 People’s Republic of China; 2 State Key Laboratory of Military Stomatology, National Clinical Research Center for Oral Diseases, Shanxxi Clinical Research Center for Oral Diseases, Department of Orthodontics, School of Stomatology, The Fourth Military Medical University, Xi’an, Shaanxi 710032 People’s Republic of China; 3 Research and Development Center for Tissue Engineering, Fourth Military Medical University, Xi’an, Shaanxi 710032 People’s Republic of China; 4 Department of Stomatology, General Hospital of Tibet Military Region, Lhasa, Tibet 850007 People’s Republic of China; 5 School and Hospital of Stomatology, Wenzhou Medical University, Wenzhou, Zhejiang 325003 People’s Republic of China

**Keywords:** Tissue engineering, Periodontal ligament stem cells, Bone marrow mesenchymal stem cells, Cell sheet, Periodontal regeneration

## Abstract

**Background:**

Tissue-engineering strategies based on mesenchymal stem cells (MSCs) and cell sheets have been widely used for periodontal tissue regeneration. However, given the complexity in periodontal structure, the regeneration methods using a single species of MSC could not fulfill the requirement for periodontal regeneration.

**Methods:**

We researched the interaction between the periodontal ligament stem cells (PDLSCs) and jaw bone marrow-derived mesenchymal stem cells (JBMMSCs), and constructed a composite cell sheet comprising both of the above MSCs to regenerate complex periodontium-like structures in nude mice.

**Results:**

Our results show that by co-culturing PDLSCs and JBMMSCs, the expressions of bone and extracellular matrix (ECM)-related genes and proteins were significantly improved in both MSCs. Further investigations showed that, compared to the cell sheet using PDLSCs or JBMMSCs, the composite stem cell sheet (CSCS), which comprises these two MSCs, expressed higher levels of bone- and ECM-related genes and proteins, and generated a composite structure more similar to the native periodontal tissue physiologically in vivo.

**Conclusions:**

In conclusion, our results demonstrate that the crosstalk between PDLSCs and JBMMSCs in cell sheets facilitate regeneration of complex periodontium-like structures, providing a promising new strategy for physiological and functional regeneration of periodontal tissue.

**Electronic supplementary material:**

The online version of this article (doi:10.1186/s13287-016-0417-x) contains supplementary material, which is available to authorized users.

## Background

Periodontitis is a common oral disease that leads to the destruction of periodontal tissues, including alveolar bone, periodontal ligament and cementum, and finally to the loosening and loss of teeth [1]. However, existing clinical treatments, such as periodontal flap operation, guided tissue regeneration, and the introduction of certain growth factors, can only clean necrotic tissue and control inflammation, and the restoration of periodontal tissue is limited [2]. Fortunately, in recent years, with the wide application and investigation of tissue engineering in the regeneration and repair of affected tissues, the investigation of stem cells and biomaterials in the biological and functional regeneration of periodontal complex tissue has attracted more attention [3]. To date, stem cells derived from different tissues have been used in animal experiments [4–7] and clinical studies [8, 9].

Since the discovery of the mesenchymal stem cells (MSCs) with multidirectional differentiation capability termed periodontal ligament stem cells (PDLSCs) by Seo et al. in 2004, many experiments have confirmed that this type of stem cell is suitable as the seed cell for the regeneration of periodontal tissue [10, 11]. However, systematic studies have indicated that the use of PDLSCs or progenitor cells from other single tooth sources only regenerate the collagen fiber structure but cannot result in a functional periodontal ligament (PDL) [5, 6]. Whereas in the regeneration experiments for skin, cardiac muscle, and corneal/lens, researchers have activated the signal channels, including the mitigation of cell death, promotion of angiogenesis, and regulation of cellular functions through the interaction between the different stem cells, with satisfactory results having been obtained [12, 13]. In other studies, researchers have co-cultured the MSCs with other types of cells, simulated the interaction of cells from different tissues in the tissue development ex vivo, and induced the occurrence of the cooperative biological behaviors such as migration, proliferation, differentiation, and regulation of cells, thereby providing huge benefits to the regeneration of blood vessels, peripheral nerves, and cartilage [14, 15]. The above research results suggest that the techniques based on the use of single cell types may not be able to meet the requirements for the regeneration of a complex structure such as periodontal tissue.

In recent years, bone marrow-derived mesenchymal stem cells (BMMSCs) have also been widely applied in the tissue-engineering repair of periodontal tissue defects [16, 17]. The research by Hughes et al. shows that this could be achieved through the migration of blood vessels to the surrounding periodontal ligament, allowing it to participate in the formation of periodontal tissue [18]. Recent studies have shown that BMMSCs can not only migrate to the periodontal tissue, and differentiate into related tissue [19], but also improve the proliferation and differentiation of PDLSCs [20]. Additionally, other studies have confirmed that jaw bone marrow-derived mesenchymal stem cells (JBMMSCs) exhibit more enhanced in situ osteogenicity of alveolar bone than stem cells derived from the ilium [21], and the bone marrow aspiration-induced pain is much less severe than that of the latter [22]. Both factors have afforded great potential for the use of the tissue-specific BMMSCs in the regeneration of periodontal tissue. Given the above, we hypothesized that the crosstalk between PDLSCs and JBMMSCs could bring benefits to periodontal regeneration.

In addition to the consideration of choice and optimization of seed cells, a strategy for cell delivery should also be emphasized in tissue-engineered regenerative medicine. The delivery of a sufficient number of seeds cells with excellent activity to the defect area [23] is the first step to ensuring the clinical feasibility of a stem cell-based treatment model [24]. Therefore, a technique called “Cell Sheet Engineering” that uses the extracellular matrix (ECM) secreted during the culture of cells as the endogenous scaffold material has been introduced to the field of periodontal tissue regeneration [25]. This technique avoids the protein damage caused by enzymatic digestion and thus is beneficial to the interaction among the cells and between cells and ECM [26]. As a result, this technique has been applied to the repair of periodontal tissue defects [5, 6]. Previously, our group conducted research on the construction method [27, 28], cell types [29, 30], application strategy [31, 32], and growth factors [20, 33] of cell sheets and achieved some results. However, the regeneration of a complete, fully physiologically functional, complex periodontal structure remains a challenge.

Considering the above research results, we have investigated the mutual effect of co-culturing the PDLSCs and JBMMSCs for the generation of osteoblasts and ECM, based on which a novel cell sheet containing MSCs derived from two different tissue sources was constructed for the regeneration of periodontal tissue. In addition, human treated dentin matrix (hTDM) and calcined bovine bone (CBB), which simulate dental matrix and alveolar bone under physiological conditions, were used as the scaffold materials for the in vivo heterotopic regeneration experiments. We hypothesized that with the interaction between the PDLSCs and JBMMSCs, this composite stem cell sheet would exhibit an enhanced ability to form periodontal-like soft and hard complex structures compared with the cell sheet based on single-variety cells, thus providing a new strategy for the treatment of periodontal tissue defects.

## Methods

### Sample collection and cell culture

hPDLSCs were obtained from the healthy periodontal ligament of premolars extracted from donors (ten donors aged 12–22 years) under orthodontic treatment. hJBMMSCs were obtained from the patients (aged 18–39 years) undergoing orthognathic surgery.

The isolation of hPDLSCs and hJBMMSCs has been described previously [10, 22]. In brief, PDL tissues were gently separated from the surface of the mid-third of the root, and subsequently digested with 3 mg/ml of collagenase type 1 (COL-1, Sigma-Aldrich, St. Louis, MO, USA) for 30 min at 37 °C. Single-cell suspensions were obtained by passing samples through a 70-μm strainer (Falcon, BD Labware, Franklin Lakes, NJ, USA). The cells were seeded into six-well plates and cultured with α-minimum essential medium (α-MEM, Gibco BRL, Gaithersburg, MD, USA) supplemented with 15 % fetal bovine serum (FBS, Gibco, BRL) and incubated in 5 % CO_2_ at 37 °C. Jaw bone debris was rinsed with α-MEM (Gibco BRL) containing 100 U/mL of penicillin and 100 mg/mL of streptomycin (Invitrogen, Carlsbad, CA, USA) three times. The debris were cut into 1 mm^3^ pieces and passed through a 70-mm strainer to obtain single-cell suspensions. Both the cell suspensions and jaw bone pieces were seeded into 10-cm dishes and cultured as hJBMMSCs. hPDLSCs and hJBMMSCs at passage three were used for the following experiments.

## Cell identification

### Colony-forming assays

Single-cell suspensions (2 × 10^3^ cells) within α-MEM (10 % FBS) were seeded in 10-cm-diameter culture dishes (Corning, Lowell, MA, USA) to assess the cell reproductive activity as previous studies [31, 32]. After 14 days of culture the cells were fixed with 4 % paraformaldehyde and stained with 1 % toluidine blue. Aggregates containing ≥ 50 cells were counted as colonies under the microscope and the numbers of colonies per well were counted. The experiment was repeated at least three times.

### Osteogenic/adipogenic differentiation

Single-cell suspensions (1 × 10^5^) within α-MEM (10 % FBS) were seeded in six-well plates. On reaching 80 % confluence, cells were cultured in α-MEM supplemented with adipogenic medium (5 % FBS, 0.5 mM 3-isobutyl-1-methylxanthine, 0.5 mM hydrocortisone, and 100 mg/l indomethacin) or osteogenic medium (5 % FBS, 100 mM dexamethasone, 50 μg/ml ascorbic acid, and 5 mM β- glycerophosphate) for another 21 or 28 days; the medium was changed every 2–3 days. Then the cells were fixed with 4 % paraformaldehyde for 20 min and stained with oil red O or 2 % alizarin red at room temperature. Finally, the cells were routinely observed and photographed under an inverted microscope [31, 32].

### Flow cytometric analysis of cell phenotype

For identification of the phenotype, approximately 5 × 10^5^ cells were incubated with PE- or FITC-conjugated monoclonal antibodies against human CD34, CD45, CD90, CD105 (eBioscience, Inc., San Diego, CA, USA), CD29, CD31 and Stro-1 (R&D Systems, Inc., Minneapolis, MN, USA) or isotype-matched control IgGs. For STRO-1 staining, cells were incubated with mouse anti-human STRO-1 for 1 h on ice. After washing with 5 % heat-inactivated FBS, cells were incubated with FITC-conjugated goat anti-mouse IgM (R&D Systems, Inc.) for an additional 30 min on ice. Cells were subjected to flow cytometric analysis with a Beckman Coulter Epics XL cytometer (Beckman Coulter, Fullerton, CA, USA) [31, 32].

## The interactions between hPDLSCs and hJBMMSCs in vitro

### Indirected co-culture of hPDLSCs and hJBMMSCs

Transwell chambers with 0.4-μm pores were used to examine the interactions of hPDLSCs and hJBMMSCs [20]. hPDLSCs/hJBMMSCs were seeded in six-well plates at a density of 2.5 × 10^5^/ml, which were covered with Transwell chambers. Correspondingly, hJBMMSCs/hPDLSCs were seeded in chambers. Then, hPDLSCs and hJBMMSCs were co-cultured reciprocally in α-MEM medium with 10 % FBS as experimental groups, while they were co-cultured with themselves respectively as control groups.

### Osteogenic differentiation

The co-cultured cells were treated with osteogenic medium for 14–28 days, then washed twice in PBS after fixation in 4 % paraformaldehyde for 30 min in 4 °C. Calcium accumulation was detected at day 28 by 2 % alizarin red staining and dissolved by 1 mL of sodium dodecyl sulfate solution. The light absorption of sodium dodecyl sulfate solution with alizarin red was read at 570 nm with a microplate reader (Bio-TEK Instruments, Inc., Winooski, VT, USA). Alkaline phosphatase (ALP) staining and activity were determined at day 14 with the BCIP/NBT Alkaline Phosphatase Color Development Kit (Beyotime Co., Shanghai, China) and Alkaline Phosphatase (AKP/ALP) Detection Kit (Zhong Sheng Co., Beijing, China) [20]. The experiment was repeated at least three times.

### Real-time polymerase chain reaction (RT-PCR) assay and Western blot (WB) assay

RNA from co-cultured cells was split, collected, purified and extracted with Trizol Reagent (Invitrogen) according to the manufacturer’s protocol. The cDNA synthesis and PCR were performed as previous described [31, 32]. The primer sequences for ALP, COL-1, bone sialoprotein (BSP), osteocalcin (OCN), fibronectin, integrinβ1, periostin and β-actin are listed in Table 1. β-actin was used as an internal control. The PCR program was 94 °C for 5 min, followed by 35 cycles of 94 °C for 45 s, 57 °C for 45 s, and 72 °C for 1 min, and a final extension step at 72 °C for 1 min. This experiment was repeated three times, and the PCR products were further confirmed by sequencing (Sangon Biotechnology Co., Shanghai, China).

**Table 1:** Primer sequences

Gene	Forward	Reverse
ALP	5′-TAAGGACATCGCCTACCAGCTC-3	5′-TCTTCCAGGTGTCAACGAGGT-3
BSP	5′-GATTTCCAGTTCAGGGCAGTAG-3′	5′-CCCAGTGTTGTAGCAGAAAGTG-3′
COL-1	5′-CCAGAAGAACTGGTACATCAGCAA-3′	5′-CGCCATACTCGAACTGGAATC-3′
FIBRONECTIN	5′-CACCCAATTCCCTTGCTGGTATC-3′	5′-TATTCGGTTCCCGGTTCCA-3′
INTEGRINβ1	5′-GTGAGTCAACCCCAACTACACT-3′	5′-AAGGCTCTGCACTGAACACATTC-3′
OCN	5′-CCCAGGCGCTACCTGTATCAA-3′	5′-GGTCAGCCAACTCGTCACAGTC-3′
PERIOSTIN	5′-GCTGCCATCACATCGGACATA-3′	5′-GCTCCTCCATAATAGACTCAGAACA-3′
RUNX2	5′-CCCGTGGCCTTCAAGGT-3′	5′-CGTTACCCGCCATGACAGTA-3′
β-ACTIN	5′-TGGCACCCAGCACAATGAA-3′	5′-CTAAGTCATAGTCCGCCTAGAGCA-3′

Total cellular protein was extracted from co-cultured cells after 7 days of culture with lysis buffer (pH 8.0) containing 1 % NP-40 (Sangon Co., Shanghai, China), 50 mM Tris(hydroxymethyl)aminomethane (Tris)-hydrogen chloride (HCl), 150 mM sodium chloride (NaCl), 0.1 mM phenylmethylsulfonyl fluoride, and 1 mg/ml aprotinin (Sigma-Aldrich) [31, 32]. We determined the protein concentration in the extracted lysates by measuring the absorbance at 595 nm with a protein assay solution (Bio-Rad, Hercules, CA, USA). Aliquots of 20–50 μg of cell lysate were separated by 10 % sodium dodecyl sulfate-polyacrylamide gel electrophoresis (SDS-PAGE) and then transferred to a polyvinylidene fluoride (PVDF) membrane (Bio-Rad). The membranes were blocked with 5 % milk for 2 h and then incubated with primary antibodies overnight. The immune complexes were incubated with horseradish peroxidase-conjugated anti-goat or anti-mouse IgG antibodies (Boshide, Beijing, China). Immunodetection was performed with the Western-Light chemiluminescent detection system (Peiqing, Shanghai, China). Primary antibodies were purchased from the following commercial sources: polyclonal antibodies against runt-related gene 2 (RUNX2), alkaline phosphatase (ALP), osteocalcin (OCN), periostin, integrinβ1, fibronectin from Abcam, (Cambridge, UK) monoclonal antibodies against bone sialoprotein (BSP) and collagen I (COL-1) from Santa Cruz Biotechnology (Dallas, TX, USA), and monoclonal antibodies against β-actin from Cell Signaling Technology (Danvers, MA, USA).

## Evaluation of three different types of cell sheets in vitro

### Construction strategy of different cell sheets

Multiple colony-derived hPDLSCs, hJBMMSCs and the mixed cells of the two in equal proportions at passage 3 were seeded at approximately 1 × 10^5^/mL into six-cell plates, and cultured in normal α-MEM medium containing 10 % FBS for 3 days (Additional file 1A). After reaching 90 % confluence, the medium was changed into the α-MEM medium, containing 10 % FBS and ascorbate (50 μg/ml). After 9–10 days, the cell sheets, including periodontal ligament stem cell sheet (PDLSCS), jaw bone marrow-derived mesenchymal stem cell sheet (JBMMSCS) and composite stem cell sheet (CSCS), were formed and easily detached from the culture plates with a cell scraper (Additional file 1B-D) [31, 32].

### Osteogenic differentiation

The attached cell sheets were washed twice in PBS after fixation in 4 % paraformaldehyde for 30 min in 4 °C. Alizarin red staining and ALP staining were performed and quantified as before. The experiment was repeated at least three times.

### Real-time polymerase chain reaction (RT-PCR) assay and Western blot (WB) assay

The expression of gene and protein (ALP, COL-1, RUNX2, BSP, OCN, periostin, fibronectin, and integrinβ1) were detected in the exactly same way as before.

### Hematoxylin and eosin (H&E) and immunohistochemical staining

The detached cell sheets were fixed in 4 % phosphate-buffered paraformaldehyde for 24 h, paraffin-embedded, longitudinally sectioned and stained with H&E as previous described [31, 32]. Other sections were incubated with primary antibodies following anti-ALP (1:200, Abcam), anti-COL-1 (1:200, Santa Cruz Biotechnology), anti-fibronectin (1:200, Abcam,), anti-periostin (1:200, Abcam), and anti-integrinβ1 (1:200, Abcam). PBS was used for the negative controls instead of the primary antibodies. Biotinylated secondary antibodies (1:1000) were purchased from Dako (Dako, Santa Clara, CA, USA). The staining sections were observed with a light microscope (Nikon, Tokyo, Japan).

### Scanning electron microscopy (SEM) observation

The detached cell sheets were fixed by 4 % paraformaldehyde. The samples were anodized in an electrolyte containing 0.5 wt% hydrofluoric acid and 1 M phosphoric acid for 1 h. After that, the whole complex was observed by scanning electron microscope (SEM) (Hitachi, S-4800, Tokyo, Japan).

## Preparation of ceramic bovine bone (CBB) and human treated dentin matrix (hTDM)

### Human treated dentin matrix preparation

The procedure for the preparation of hTDMs was described previously [30]. Briefly, premolar teeth, removed for clinical reasons at the School of Stomatology, Fourth Military Medical University, were collected. Periodontal tissue was completely scraped away using a curette along with removal of the outer cementum and part of the dentin. Dental pulp tissues and pre-dentin were also mechanically removed. For the fabrication of hTDM, the dentin matrix was formed to a length of 5.0 mm and a thickness of up to 2.0 mm and mechanically cleaned using an ultrasonic cleaner. Human dentin matrices were then treated with 17 % ethylenediaminetetraacetic acid (EDTA, Sigma-Aldrich) for 5 min, 10 % EDTA for 5 min, 5 % EDTA for 10 min. hTDMs were maintained in sterile PBS with 100 unit/ml penicillin (Hyclone, Logan, UT, USA) and 100 mg/ml streptomycin (Hyclone) for 72 h, then washed in sterile deionized water for 10 min in an ultrasonic cleaner, finally stored in α-MEM at 4 °C.

Finally, the mixed cells, including hPDLSCs and hJBMMSCs, were seeded onto the prepared hTDM and incubated in normal α-MEM medium containing 10 % FBS for 7 days to inspect the cellular compatibility of the scaffold.

### Ceramic bovine bone preparation

The ceramic bovine bone (CBB) was produced from fresh bovine rib bones, which was subsequently cut into blocks, washed in normal saline and soaked in H_2_O_2_ to remove proteins. Next, the blocks were formed to a length of 5.0 mm and a thickness of up to 2.0 mm. These blocks were washed with running water, heated at 900 °C for 1 h, and sterilized in a high temperature and pressure environment. Then they were washed in sterile deionized water for 10 min in an ultrasonic cleaner, finally stored in α-MEM at 4 °C [20].

Finally, the mixed cells, including hPDLSCs and hJBMMSCs, were seeded onto the prepared CBB and incubated in normal α-MEM medium containing 10 % FBS for 7 days to inspect the cellular compatibility of the scaffold.

### Observation of the hTDM/CBB surface via scanning electron microscopy) analysis

The hTDM/CBB with/without cells was fixed by 4 % paraformaldehyde. The samples were anodized in an electrolyte containing 0.5 wt% hydrofluoric acid and 1 M phosphoric acid for 1 h. After that, the whole complex was observed by scanning electron microscope (SEM) (Hitachi, S-4800) [31, 32].

## In vivo transplantation

### Nude mice ectopic transplantation

The hTDMs/CBBs coated respectively by different types of cell sheets were kept in the wells of 12-well plates with a minimal amount of cell culture medium as grafts (Additional file 1E, F). Eighteen 6-week-old immunodeficient mice (BALB/c-nu; FMMU Medical Laboratory Animal Center, Xi’an, China) were used in this experiment. Mice were anesthetized, and the dermal space was created by blunt lateral dissection from a single dorsal midline incision. Each mouse received two grafts, one on each side. The wounds were sutured to achieve primary closure (Additional file 1G) [31, 32]. Six weeks after transplantation, the mice were euthanized, and the grafts were removed for histological analysis.

### Morphological and histomorphometric evaluation of regenerated PDL tissues

The samples were fixed in 4 % paraformaldehyde overnight at 4 °C, decalcified with 17 % EDTA (pH 8.0), embedded in paraffin, and cut into 5-μm sections. For histological analysis, sections were stained with hematoxylin and eosin (H&E) and Masson’s Trichrome as previous described [31, 32]. Deparaffinized sections were immersed in 3 % H_2_O_2_/methanol for 15 min to quench endogenous peroxidase activity and incubated with primary antibodies (1:100 to 1:500 dilutions) overnight at 4 °C. Polyclonal antibodies against human cementum protein 1 (CEMP1) and BSP from Abcam were used as primary antibodies. Isotype-matched control antibodies were used under the same conditions as the primary antibodies. Sections were counterstained with hematoxylin. A microscope (Leica Microsystems AG, Wetzlar, Germany) was used for histological evaluation, which was based on the morphological observation of three sections per complex.

### Statistical analysis

All results are representative of data generated in three independent experiments. All of the statistical analyses were performed using ANOVA followed by Fisher’s protected least significant difference (PLSD) test and Student’s *t* test by SPSS version 15.0 software (SPSS, Inc., Chicago, IL, USA). All of the values are expressed as mean ± SD. A *P* value <0.05 was considered to be statistically significant. All procedures were performed blind.

## Results

### Isolation and characterization of hPDLSCs and hJBMMSCs

**Fig. 1:**
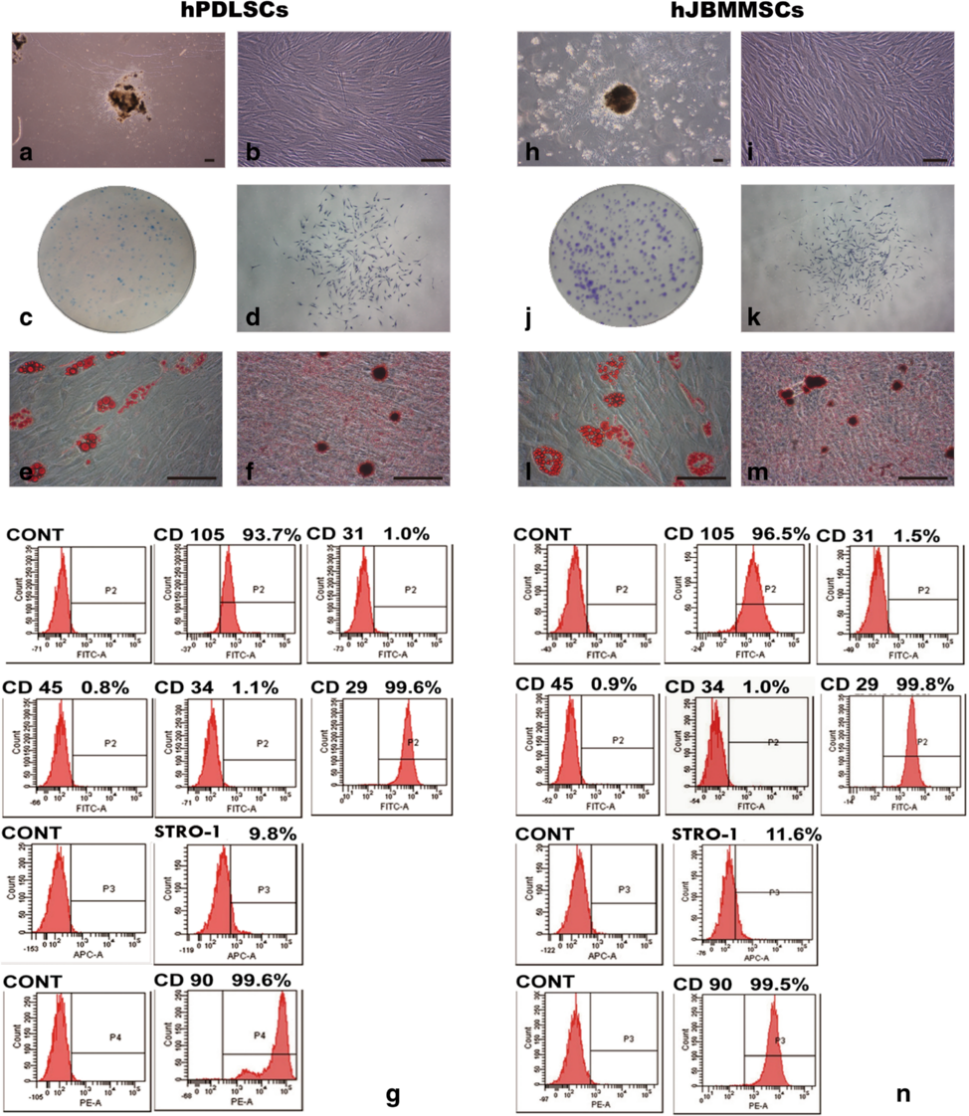
Sample collection and characterization of hPDLSCs/hJBMMSCs. **a**, **h** Primary human PDLSCs and JBMMSCs. **b**, **i** hPDLSCs/hJBMMSCs grown in culture medium, both showing the long spindle shape. **c**, **d**, **j**, **k** Representative figures showed the proliferation of a single clone of hPDLSCs/hJBMMSCs. **e**, **l** Cultured hPDLSCs/hJBMMSCs formed oil red O-positive lipid clusters following 21 days of adipogenic induction. **f**, **m** When hPDLSCs/hJBMMSCs were cultured in osteogenic inductive conditions for 21 days, mineralized nodules were found by alizarin red S staining. **g**, **n** Flow cytometric analysis of ex vivo-expanded hPDLSCs/hJBMMSCs revealed positive expression of STRO-1, CD105, CD90, CD29, and negative expression of CD31, CD34 and CD45. The scale bar represents 25 μm. *hJBMMSCs* human jaw bone marrow-derived mesenchymal stem cells, *hPDLSCs* human periodontal ligament stem cells

The primary hPDLSCs/hJBMMSCs were seen around the tissue pieces (Fig. 1a, h), and retained their fibroblast spindle shape after passage (Fig. 1b, i). When they were cultured at a low density, they formed adherent clonogenic cell clusters (colony-forming unit, fibroblastic, CFU-F) (Fig. 1d, k). The colony formation efficiency of hPDLSCs/hJBMMSCs was 27.3 % and 25.7 % respectively (Fig. 1c, j).

After culturing in adipogenesis-inducing medium for 21 days, hPDLSCs/hJBMMSCs both were observed by oil red staining and were found to form lipid droplets (Fig. 1e, l). After induction in osteogenesis medium for 21 days, they were both observed with alizarin red staining and were found to form mineralized nodules (Fig. 1f, m).

hPDLSCs/hJBMMSCs both exhibited a characteristic pattern of mesenchymal surface markers, including CD90, CD105, CD29 and STRO-1, whereas the hematopoietic markers CD31, CD34 and CD45 were negative (Fig. 1g, n).

### The crosstalk between hPDLSCs and hJBMMSCs in vitro

**Fig. 2:**
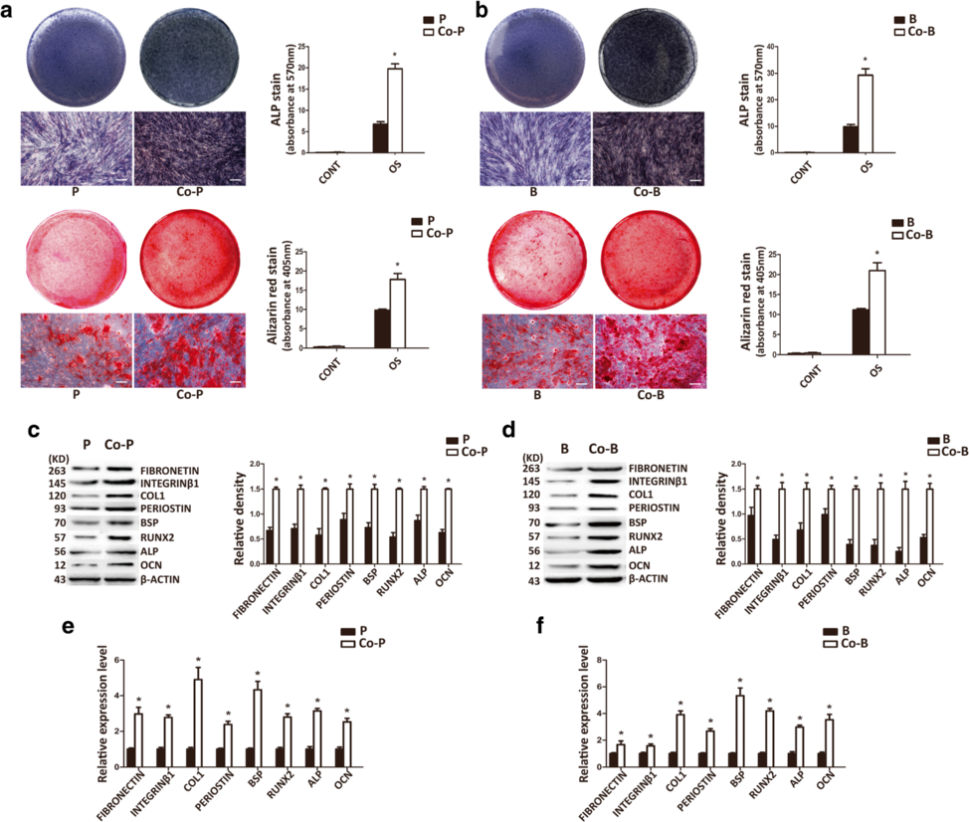
The crosstalk between hPDLSCs and hJBMMSCs in vitro. **a**, **b** Osteogenic differentiation/ALP activity and their quantitative results of co-cultured hPDLSCs/hJBMMSCs and hPDLSCs/hJBMMSCs by alizarin red staining and ALP staining. **c**, **d** The results of Western blot and quantitation show the expression of osteoblast- and ECM-related proteins in co-cultured hPDLSCs/hJBMMSCs and hPDLSCs/hJBMMSCs. **e**, **f** The results of PCR show the expression of osteoblast- and ECM-related genes in co-cultured hPDLSCs/hJBMMSCs and hPDLSCs/hJBMMSCs. *P* hPDLSCs, *Co-P* co-cultured hPDLSCs, *B* hJBMMSCs, *Co-B* co-cultured hJBMMSCs. The data are shown as mean ± SD. ^*^ *P* < 0.05, n = 3. The scale bar represents 50 μm

To investigate the mutual effect between hPDLSCs and hJBMMSCs in osteogenic capability, they were co-cultured by Transwell method in osteogenic differentiation media. The results of alizarin red staining and ALP staining both showed that the co-cultured stem cells could form more mineralization nodules and exhibit higher ALP activity, compared with corresponding controls (Fig. 2a, b). To further analyze, real-time PCR was conducted, which demonstrated that the osteogenesis- and ECM-related gene expression of ALP, COL-1, RUNX2, BSP, OCN, fibronectin, integrinβ1, and periostin was much higher in co-cultured cells than cells in control groups (Fig. 2e, f). Meanwhile, the same trend was observed in the protein expression levels (Fig. 2c, d). These data indicated that the interactions between hPDLSCs and hJBMMSCs might promote their osteogenic differentiation potential and capability of ECM formation.

### Evaluation of three different types of cell sheets in vitro

**Fig. 3:**
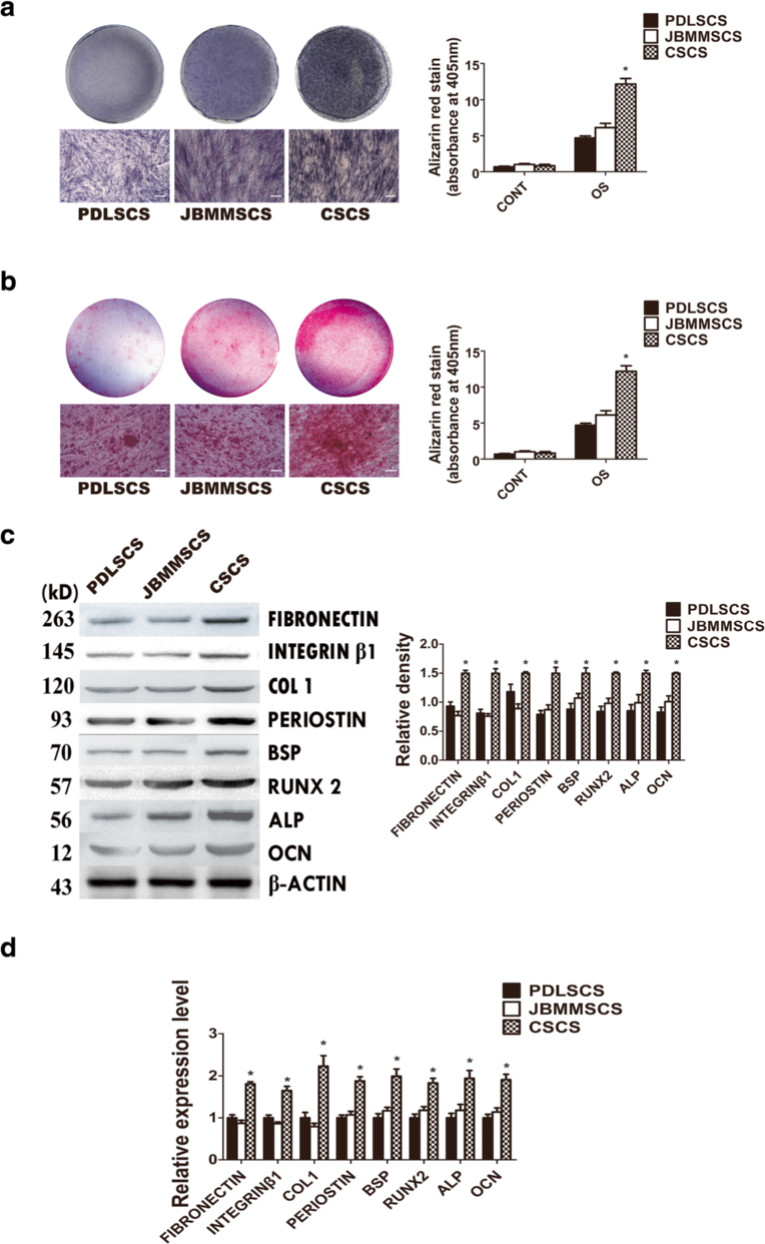
Investigation of the difference of PDLSCS, JBMMSCS, and CSCS in vitro. **a** Osteogenic differentiation of the three types of cell sheets assessed by alizarin red staining and quantified by absorptiometry. **b** ALP activity of the three types of cell sheets assessed by ALP staining and quantified by absorptiometry. **c** The results of Western blot and quantitation show the expression of osteoblast- and ECM-related proteins in the three types of cell sheets. **d** The results of PCR show the expression of osteoblast- and ECM-related genes in the three types of cell sheets. *PDLSCS* periodontal ligament stem cell sheet, *JBMMSCS* jaw bone marrow-derived mesenchymal stem cell sheet, *CSCS* composite stem cell sheet. The data are shown as mean ± SD. ^*^ *P* < 0.05, n = 3. The scale bar represents 50 μm

To investigate the difference of PDLSCS, JBMMSCS, and CSCS in osteogenic capability, they were cultured in osteogenic differentiation media. The results of alizarin red staining and ALP staining both showed that CSCS could form more mineralization nodules and exhibit higher ALP activity, compared with the other two (Fig. 3a, b).

Furthermore, real-time PCR showed that the osteogenesis- and ECM-related genes expression of ALP, COL-1, RUNX2, BSP, OCN, Fibronectin, Integrinβ1, and Periostin, was much higher in CSCS than PDLSCS/JBMMSCS (Fig. 3d). And the same trend was observed in the protein expression levels (Fig. 3c).

**Fig. 4:**
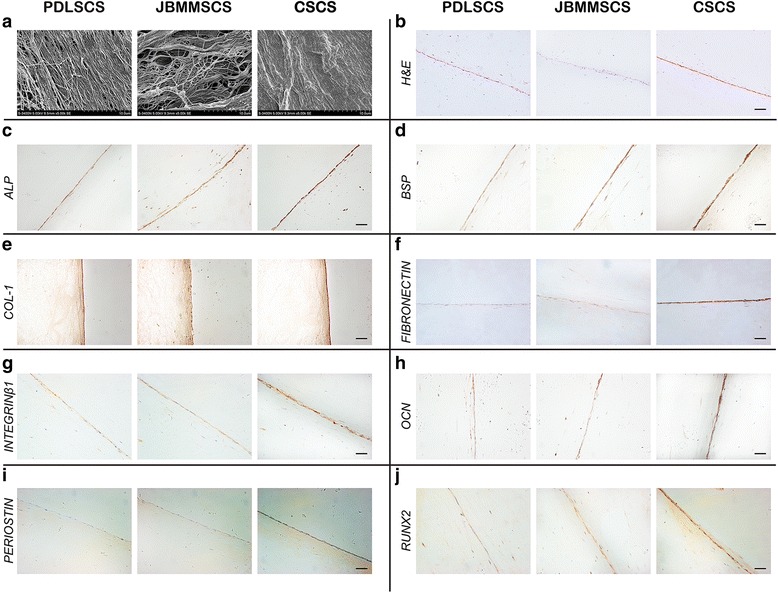
Morphological characteristics and immunohistochemical analyses of PDLSCS, JBMMSCS, and CSCS. **a** The arrangement of cells in all three cell sheets was observed by SEM. **b** H&E staining showed all three cell sheets were dense and contained plenty of cells, which could ensure collagen secretion. **c**-**j** All three cell sheets positively expressed ALP, BSP, COL-1, OCN, RUNX2, fibronectin, periostin and integrinβ1. ALP, BSP, OCN, RUNX2 and periostin indicated the osteogenic differentiation capacity. COL-1, integrinβ1, and fibronectin were the markers of ECM. *PDLSCS* periodontal ligament stem cell sheet, *JBMMSCS* jaw bone marrow-derived mesenchymal stem cell sheet, *CSCS* composite stem cell sheet. The scale bar represents 100 μm

Additionally, the SEM analysis demonstrated all three cell sheets established a film-like cell network that retained tight junctions between cells, while among the three cell sheets, CSCS contained the most compacted cell arrangement and collagen secretion (Fig. 4a). H&E staining and immunohistochemical staining showed that all three cell sheets were dense and contained plenty of cells (Fig. 4b), and all three cell sheets positively expressed ALP (Fig. 4c), BSP (Fig. 3d), COL-1 (Fig. 4e), fibronectin (Fig. 4f), integrinβ1 (Fig. 4g), OCN (Fig. 4h), periostin (Fig. 4i) and RUNX2 (Fig. 4j), while the staining intensity of all the proteins in CSCS increased notably, compared with the other two cell sheets. These data suggested that the interactions between different kinds of cells during the formation of cell sheets may result in CSCS possessing more capability in osteogenic differentiation and ECM secretion than other two cell sheets.

### Regenerative PDL-like tissue formation around scaffolds surface in vivo

CBB and hTDM were chosen to simulate the two kinds of hard tissues, which are anatomically located on either side of the periodontal membrane in normal physiological conditions. SEM analysis of the scaffold CBB showed a porous structure (Additional file 2A, B). In contrast, the surface of hTDM showed dentinal tubules were sufficiently exposed after being treated with EDTA (Additional file 2E, F). The SEM images also showed that both hPDLSCs and hJBMMSCs could adhere to the scaffolds well, proliferate adequately, and extend excessively on the surface of hTDM and CBB (Additional file 2C, D, G, H).

**Fig. 5:**
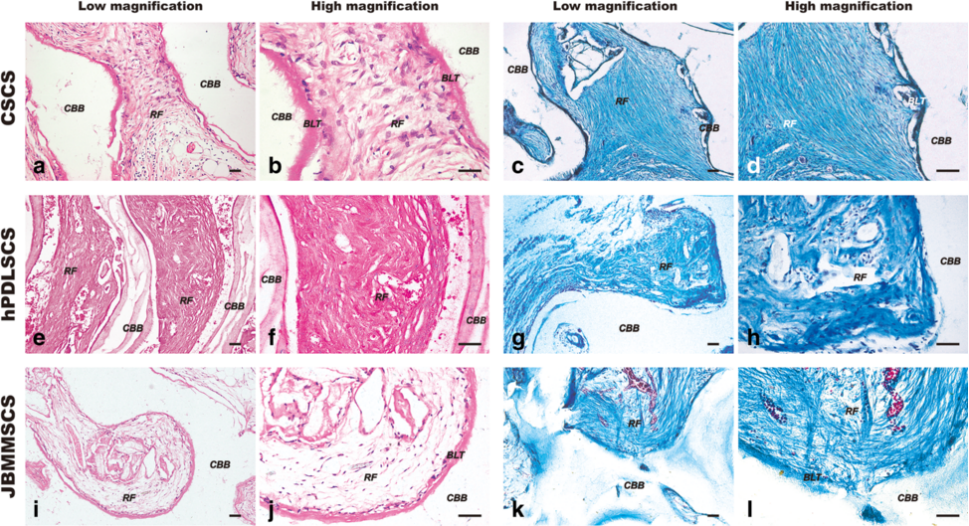
Regeneration of PDL/bone-like tissue around the CBB in nude mice. **a**-**d** More bone-like tissue and inserting PDL-like fibers around the CBB were observed in the CSCS group by H&E and Masson tricolor staining. **e**-**h** Plenty of cluttered fibers were formed in the PDLSCS group. **i**-**l** Bone-like tissue and sparse and unordered fibers around CBB were observed in the JBMMSCS group. *BLT* bone-like tissue, *CBB* ceramic bovine bone, *RF* regenerated fibers. *PDLSCS* periodontal ligament stem cell sheet, *JBMMSCS* jaw bone marrow-derived mesenchymal stem cell sheet, *CSCS* composite stem cell sheet, *CBB* ceramic bovine bone. The scale bar represents 100 μm

At the end of 6 weeks after transplantation of the cell sheets into the subcutaneous space of the immunodeficient mice, we harvested all 36 regenerated tissue specimens and examined their morphology. H&E and Masson Trichrome staining showed that an amount of dense, ordered fibers were closely adhered to the surface of the CBB, with notable mineralized deposition, in the CSCS group (Fig. 5a-d). Whereas plenty of cluttered fibers were formed in the PDLSCS group (Fig. 5e-h), and in the JBMMSCS group, the regenerated fibers were sparse and unordered, although there was a deposition of osteoids (Fig. 5i-l).

**Fig. 6:**
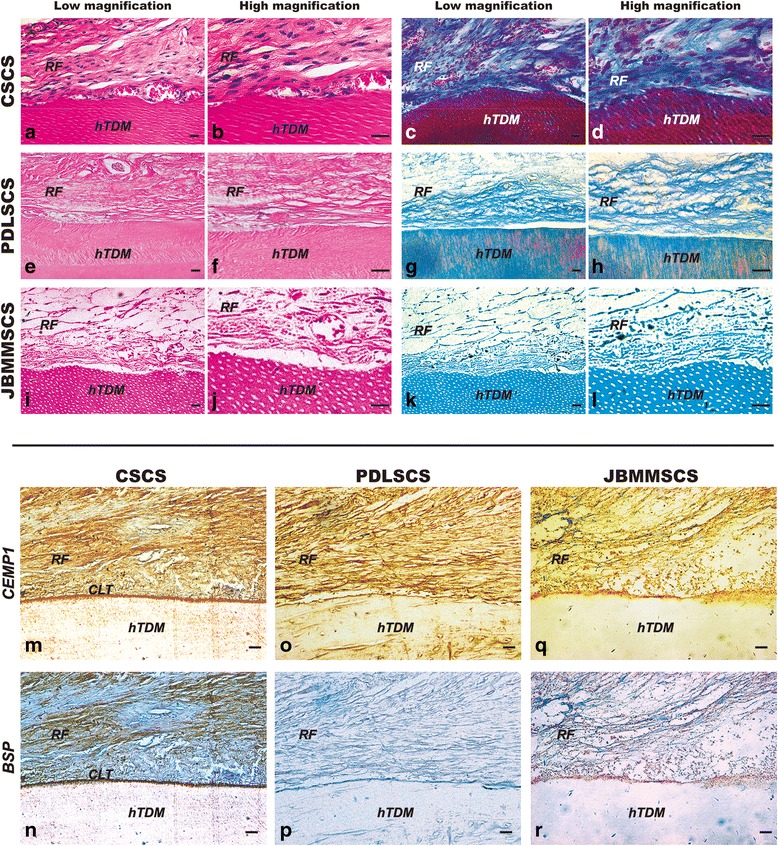
Regeneration of PDL/cementum-like tissue around hTDM in nude mice. **a**-**d** Typical arranged tissue with Sharpey-like fibers was regenerated, which was closely attached to the surface of hTDM at an oblique or horizontal angle in the CSCS group by H&E and Masson tricolor staining. **e**-**h** Few inserted fibers were observed, and most of fibers were parallel with the surface of the scaffolds in the PDLSCS group. **i**-**l** A few collagenous fibers, sparsely and chaotically arranged, in regenerated tissues in the JBMMSCS group. **m**, **n** Immunohistochemical staining showed high expression of CEMP1 and BSP in the CSCS group, especially in the juncture of the soft and hard tissues. **o**, **p** The expression of CEMP1 was positive, while the expression of BSP was negative in the PDLSCS group. **q**, **r** The low expressions of CEMP1 and BSP were observed in the JBMMSCS group. *CLT* cementum-like tissue, *hTDM* human treated dentin matrix, *RF* regenerated fibers. *PDLSCS* periodontal ligament stem cell sheet, *JBMMSCS* jaw bone marrow-derived mesenchymal stem cell sheet, *CSCS* composite stem cell sheet, *hTDM* human treated dentin matrix. The scale bar represents 100 μm

On the other hand, typical arranged tissue with Sharpey-like fibers was regenerated, which was closely attached to the surface of hTDM at an oblique or horizontal angle in the CSCS group (Fig. 6a-d). In contrast, in the PDLSCS group, few inserted fibers were observed, and most of the fibers were parallel with the surface of the scaffolds (Fig. 6e-h), and in the JBMMSCS group, there were a few collagenous fibers, sparsely and chaotically arranged, in regenerated tissues (Fig. 6i-l). Furthermore, immunohistochemical staining showed that the expressions of CEMP1 and BSP were much higher in the CSCS group (Fig. 6m, n) than in the two other groups (Fig. 6o-r), especially in the juncture of soft and hard tissues. Taken together, the CSCS, which regenerated PDL-like tissue not only on the CBB, but also on the hTDM, may be more appropriate for periodontal regeneration than the PDLSCS/JBMMSCS.

## Discussion

In recent years, to overcome the inability of traditional periodontal treatment methods to regenerate functional periodontal tissue [1], the tissue-engineering repair of periodontal tissue based on the use of stem cells has received widespread attention and been the subject of more in-depth investigations [3]. Furthermore, the cell sheet technique, a cell delivery strategy that retains the autocrine function of the ECM in the same cells, has been incorporated into more and more applications in the study of tissue-engineering repair of periodontal tissue [25, 34], and cell sheets constructed of cells derived from different tissues have been used in the repair of periodontal tissue defects in animals, and certain effects have been reported [5, 35]. However, the use of a single type of cell cannot reliably regenerate periodontal tissue, a complex structure containing both soft and hard tissues. Among the stem cells that have been used in the regeneration of periodontal tissue, the PDLSCs and BMMSCs have attracted more attention for their potential in the regeneration engineering of periodontal tissue because of their strong proliferation capability and ability to differentiate into periodontal tissue, as well as their wide availability [3, 23]. In addition, previous research has also found that the MSCs derived from the jaw exhibited a stronger potential to form periodontal complex tissue than MSCs derived from the ilium [21, 36]. Therefore, the present study utilized PDLSCs and JBMMSCs, two types of MSCs derived from tissues closely related to periodontal tissue. In addition, the interaction between these two types of MSCs has been investigated, and a novel cell sheet comprising these two, which are derived from different tissues, has been constructed. This composite stem cell sheet, which exhibits unique biological characteristics, is capable of forming complex periodontal-like soft and hard tissue structures in subcutaneous areas of nude mice, providing a new possibility for the treatment of periodontal tissue defects.

The regeneration of functional periodontal-like tissue that contains both soft and hard tissues is the ultimate goal of the tissue-engineering repair of periodontal tissue defects. Among these, the regeneration of bone tissue in specific locations is particularly important because not only does bone tissue provide the most important structural basis for the stability of the tooth root, but it also generates Sharpey’s fiber. This fiber is a unique dense connective tissue in periodontal tissue that perforates soft and hard tissues and connects the cementum of the tooth root to the alveolar bone. This connective tissue is required for the proper functioning of periodontal tissue. Similar to the formation of bone tissues in other locations, the bone matrix-related proteins that are synthesized and secreted by the same cells are the basis for the accumulation of calcium and thus the mineralization of tissue [37]. Therefore, in this experiment, we measured the levels of ALP, OCN, BSP, RUNX2, and periostin, which are expressed in normal periodontal tissue and participate in the mineralization and osteogenesis of tissue at different stages from the cellular level and the cell sheet level. The results showed that the expression of the genes and proteins for the aforementioned materials was higher than that in the control groups. The staining of the calcium nodule, the activity of the ALP, and the immunohistochemistry of paraffin sections of the cell sheet (IHC-P) also confirmed this finding. Therefore, we believe that the PDLSCs and JBMMSCs can enhance each other’s capacity for osteogenic differentiation. Moreover, compared to the cell sheets comprising a single cell type, the cell sheet that comprises these two types of stem cells shows stronger osteogenic potential, which is beneficial to the functional regeneration of periodontal tissue. Furthermore, the expression of the ECM-related genes and proteins in the experimental group, including the COL-1, fibronectin, integrinβ1, that closely participate in cell adhesion, migration, proliferation, differentiation, and apoptosis [38] was significantly increased, suggesting that the cell sheet constructed from the indirect or direct co-culture exhibited stronger ability to synthesize and secrete ECM. In the cell sheet technology, the ECM secreted by the same cells forms a biochemical and mechanical cell niche. The various factors within the cell niche affect the cellular activity; coordinate the interactions among the cells, the soluble cellular factors, and the ECM [39]; and regulate the structure and function of the newly regenerated tissue. Therefore, enhancing the formation and preservation ability of the ECM by seed cells is beneficial to the complete regeneration of the periodontal tissue.

In the regeneration of complex tissues, the importance of microenvironment in the regulation of seed cells has been confirmed [40]. The various cellular factors in the microenvironment can enhance the regeneration of complex tissues by affecting the migration, proliferation, and differentiation of the stem cells [41, 42]. However, a continuous and effective stimulation of the seed cells is often lacking in the microenvironment of the in vivo defects or in the areas requiring regeneration. The factors secreted by a single cell type are limited and cannot completely improve the microenvironment in the defect locations, thus resulting in pronounced apoptosis [43], deficiency of vascularization [12], or incompleteness of the structure of the regenerated tissue [44–46]. Therefore, the construction of a physiologically similar microenvironment to that which produces and maintains the periodontal ligament is important. Previous studies have focused on the change in the gene expression and protein secretion of the seed cells through gene transfection [47] or on the provision of beneficial environments to seed cells by using the cell culture supplements derived from plasma that is rich in growth factors [48] or conditioned medium derived from embryonic cells [49]. However, all these methods have limitations. We found that the results of previous studies suggest that the progenitor cells in the periodontal ligament might come from the marrow cavity and reach the periodontal tissue via the circulation of the blood [50]. The MSCs in the periodontal ligament on the alveolar side showed stronger multidirectional differentiation capability and expressed a higher level of osteogenesis-related proteins than those on the cement side [51]. Taking into account the intimate affinity of the two tissues [52] and their interaction in the development process [53], we speculated that the interaction between the PDLSCs and JBMMSCs plays an important role in the formation and stability of the unique microenvironment of the periodontal tissue. Therefore, in this study, we directly mixed these two types of MSCs to construct the cell sheet and reconstruct the interaction of the two types of stem cells under physiological conditions, while simultaneously forming an ECM that contained multiple factors beneficial to in vivo regeneration. Compared to previous methods, the stable and direct relation among the cells established through co-culture gives rise to a more sustainable, comprehensive, and safe simulation and effect on the target cells. In addition, the resultant change in the gene and protein levels compensates for the signal molecules that were lost in the ex vivo culture [54]. All of these factors positively contribute to the regeneration of complex tissues. The in vivo heterotopic regeneration experiments in nude mice also confirmed our hypothesis. Whether on the surface of hTDM or CBB, the composite cell sheet comprising two types of stem cells formed a large amount of a dense and ordered periodontal-like composite structure that closely connects the osteoid and scaffolding materials, with high expression of CEMP1 and BSP, which play vital roles in the growth and development of periodontal and cementum tissue [3, 55]. However, the regenerated tissue from a single cell type exhibited defects in fiber density, direction, and the accumulation of new bone. Besides, the species reactivity of the antibodies used in our experiments is human, which indicates the origin of regenerated tissues.

Although we have observed the biologically functional benefits from the interaction of the MSCs in the ex vivo experiment and successfully regenerated periodontal-like composite tissue structure in the in vivo heterotopic experiment, some problems remain to be resolved, e.g., the mechanism of the interaction between the two cell types, and the fate of the implanted cells in vivo. Recent studies on exosomes [56] and Gabriel Rahmi’s latest study [57] revealed that although most of the implanted cells would die in a few days, they could be involved in tissue repair through the paracrine effect, which offer us some insights regarding future investigations. In addition, clinical periodontal tissue defects normally are accompanied by inflammation, whether the composite can properly function in this microenvironment remains unknown. These questions will be the focus in our future studies.

## Conclusions

In summary, the composite cell sheet comprising two types of MSCs derived from two closely related tissues provide a more suitable microenvironment for the regeneration of periodontal tissue with complex structure. This structure arises from the interaction of the two types of MSCs, thus providing a promising new strategy for the clinical repair of periodontal tissue defects.

## Additional files

Additional file 1: Construction strategy of different cell sheets and nude mouse ectopic transplantation. (A) Multiple colony-derived hPDLSCs, hJBMMSCs, and the mixed cells of the two above in equal proportions were seeded into six-cell plates, and after induction by conditioned media, (B) PDLSCS, (C) JBMMSCS, and (D) CSCS were formed. (E, F) CBB/hTDM wrapped by cell sheets was cultured as the transplantation grafts. (G) Each mouse received two grafts, one on each side. The wounds were sutured to achieve primary closure. (PDF 596 kb)
Additional file 2: Microscopic appearance of the scaffold materials and the adhesion of the cells to the scaffolds. (A, B) CBB showed a porous structure, and (E, F) the surface of hTDM showed dentinal tubules were sufficiently exposed. (C, D, G, H) The hPDLSCs and hJBMMSCs could adhere to these two scaffolds well, proliferate adequately, and extend excessively on the surface of CBB and hTDM. (PDF 511 kb)

## Abbreviations

ALP: alkaline phosphatase
BMMSCs: bone marrow-derived mesenchymal stem cells
BSP: bone sialoprotein
CBB: ceramic bovine bone
CEMP1: cementum protein 1
COL-1: collagen I
CSCS: composite stem cell sheet
ECM: extracellular matrix
EDTA: ethylenediaminetetraacetic acid
FBS: fetal bovine serum
H&E: hematoxylin and eosin
HCl: hydrogen chloride
hTDM: human treated dentin matrix
IHC-P: immunohistochemistry paraffin protocol
JBMMSCs: jaw bone marrow-derived mesenchymal stem cells
JBMMSCS: jaw bone marrow-derived mesenchymal stem cell sheet
MSCs: mesenchymal stem cells
NaCl: sodium chloride
OCN: osteocalcin
PDLSCS: periodontal ligament stem cell sheet
PDLSCs: periodontal ligament stem cells
PVDF: polyvinylidene fluoride
RT-PCR: real-time polymerase chain reaction
RUNX2: runt-related gene 2
SDS-PAGE: sodium dodecyl sulfate-polyacrylamide gel electrophoresis
SEM: scanning electron microscopy
WB: Western blot
α-MEM: α-minimum essential medium

## References

[1] Pihlstrom BL, Michalowicz BS, Johnson NW. Periodontal diseases. The Lancet. 2005;366(9499):1809-20. 10.1016/S0140-6736(05)67728-8 16298220

[2] Chen FM, Zhang J, Zhang M, An Y, Chen F, Wu ZF (2010). A review on endogenous regenerative technology in periodontal regenerative medicine. Biomaterials..

[3] Izumi Y, Aoki A, Yamada Y, Kobayashi H, Iwata T, Akizuki T, Suda T, Nakamura S, Wara-Aswapati N, Ueda M, Ishikawa I (2011). Current and future periodontal tissue engineering. Periodontology.

[4] Uematsu K, Kawase T, Nagata M, Suzuki K, Okuda K, Yoshie H, Burns DM, Takagi R (2013). Tissue culture of human alveolar periosteal sheets using a stem-cell culture medium (MesenPRO-RS): in vitro expansion of CD146-positive cells and concomitant upregulation of osteogenic potential in vivo. Stem Cell Res..

[5] Tsumanuma Y, Iwata T, Washio K, Yoshida T, Yamada A, Takagi R, Ohno T, Lin K, Yamato M, Ishikawa I, Okano T, Izumi Y (2011). Comparison of different tissue-derived stem cell sheets for periodontal regeneration in a canine 1-wall defect model. Biomaterials..

[6] Ding G, Liu Y, Wang W, Wei F, Liu D, Fan Z, An Y, Zhang C, Wang S (2010). Allogeneic periodontal ligament stem cell therapy for periodontitis in swine. Stem Cells..

[7] Requicha JF, Viegas CA, Munoz F, Azevedo JM, Leonor IB, Reis RL, Gomes ME (2014). A tissue engineering approach for periodontal regeneration based on a biodegradable double-layer scaffold and adipose-derived stem cells. Tissue Eng A..

[8] Yamada Y, Ueda M, Hibi H, Baba S (2006). A novel approach to periodontal tissue regeneration with mesenchymal stem cells and platelet-rich plasma using tissue engineering technology: a clinical case report. Int J Periodontics Restorative Dent..

[9] Feng F, Akiyama K, Liu Y, Yamaza T, Wang TM, Chen JH, Wang BB, Huang GT, Wang S, Shi S (2010). Utility of PDL progenitors for in vivo tissue regeneration: a report of 3 cases. Oral Dis..

[10] Seo BM, Miura M, Gronthos S, Bartold PM, Batouli S, Brahim J, Young M, Robey PG, Wang CY, Shi S (2004). Investigation of multipotent postnatal stem cells from human periodontal ligament. Lancet..

[11] Shi S, Bartold PM, Miura M, Seo BM, Robey PG, Gronthos S (2005). The efficacy of mesenchymal stem cells to regenerate and repair dental structures. Orthod Craniofac Res..

[12] Caspi O, Lesman A, Basevitch Y, Gepstein A, Arbel G, Habib IH, Gepstein L, Levenberg S (2007). Tissue engineering of vascularized cardiac muscle from human embryonic stem cells. Circ Res..

[13] Trottier V, Marceau-Fortier G, Germain L, Vincent C, Fradette J (2008). IFATS collection: Using human adipose-derived stem/stromal cells for the production of new skin substitutes. Stem Cells..

[14] Meretoja VV, Dahlin RL, Kasper FK, Mikos AG (2012). Enhanced chondrogenesis in co-cultures with articular chondrocytes and mesenchymal stem cells. Biomaterials..

[15] Mehnert JM, Kisch T, Brandenburger M (2014). Co-culture systems of human sweat gland derived stem cells and peripheral nerve cells: an in vitro approach for peripheral nerve regeneration. Cell Physiol Biochem..

[16] Hasegawa N, Kawaguchi H, Hirachi A, Takeda K, Mizuno N, Nishimura M, Koike C, Tsuji K, Iba H, Kato Y, Kurihara H (2006). Behavior of transplanted bone marrow-derived mesenchymal stem cells in periodontal defects. J Periodontol..

[17] Kramer PR, Kramer SF, Puri J, Grogan D, Guan G (2009). Multipotent adult progenitor cells acquire periodontal ligament characteristics in vivo. Stem Cells Dev..

[18] Hughes FJ, Turner W, Belibasakis G, Martuscelli G (2006). Effects of growth factors and cytokines on osteoblast differentiation. Periodontology.

[19] Zhou J, Shi S, Shi Y, Xie H, Chen L, He Y, Guo W, Wen L, Jin Y (2011). Role of bone marrow-derived progenitor cells in the maintenance and regeneration of dental mesenchymal tissues. J Cell Physiol..

[20] Xie H, Liu H (2012). A novel mixed-type stem cell pellet for cementum/periodontal ligament-like complex. J Periodontol..

[21] Aghaloo TL, Chaichanasakul T, Bezouglaia O, Kang B, Franco R, Dry SM, Atti E, Tetradis S (2010). Osteogenic potential of mandibular vs. long-bone marrow stromal cells. J Dent Res.

[22] Matsubara T, Suardita K, Ishii M, Sugiyama M, Igarashi A, Oda R, Nishimura M, Saito M, Nakagawa K, Yamanaka K, Miyazaki K, Shimizu M, Bhawal UK, Tsuji K, Nakamura K, Kato Y (2005). Alveolar bone marrow as a cell source for regenerative medicine: differences between alveolar and iliac bone marrow stromal cells. J Bone Miner Res..

[23] Chen FM, Sun HH, Lu H, Yu Q (2012). Stem cell-delivery therapeutics for periodontal tissue regeneration. Biomaterials..

[24] Kelm JM, Fussenegger M (2010). Scaffold-free cell delivery for use in regenerative medicine. Adv Drug Deliv Rev..

[25] Ishikawa I, Iwata T, Washio K, Okano T, Nagasawa T, Iwasaki K, Ando T (2009). Cell sheet engineering and other novel cell-based approaches to periodontal regeneration. Periodontology.

[26] Yang J, Yamato M, Shimizu T, Sekine H, Ohashi K, Kanzaki M, Ohki T, Nishida K, Okano T (2007). Reconstruction of functional tissues with cell sheet engineering. Biomaterials..

[27] Yang Z, Jin F, Zhang X, Ma D, Han C, Huo N, Wang Y, Zhang Y, Lin Z, Jin Y (2009). Tissue engineering of cementum/periodontal-ligament complex using a novel three-dimensional pellet cultivation system for human periodontal ligament stem cells. Tissue Eng Part C Methods..

[28] Yang ZH, Jin F, Zhang XJ, Liu X, Zhang YF, Liu JQ, Duan YZ, Jin Y (2010). A novel possible strategy based on self-assembly approach to achieve complete periodontal regeneration. Artif Organs..

[29] Guo W, Chen L, Gong K, Ding B, Duan Y, Jin Y (2012). Heterogeneous dental follicle cells and the regeneration of complex periodontal tissues. Tissue Eng A..

[30] Guo W, He Y, Zhang X, Lu W, Wang C, Yu H, Liu Y, Li Y, Zhou Y, Zhou J, Zhang M, Deng Z, Jin Y (2009). The use of dentin matrix scaffold and dental follicle cells for dentin regeneration. Biomaterials..

[31] Zhu B, Liu W, Zhang H, Zhao X, Duan Y, Li D, Jin Y (2015). Tissue-specific composite cell aggregates drive periodontium tissue regeneration by reconstructing a regenerative microenvironment. J Tissue Eng Regen Med.

[32] Na S, Zhang H, Huang F, Wang W, Ding Y, Li D, Jin Y (2016). Regeneration of dental pulp/dentine complex with a three-dimensional and scaffold-free stem-cell sheet-derived pellet. J Tissue Engin Regen Med.

[33] Shang F, Ming L, Zhou Z, Yu Y, Sun J, Ding Y, Jin Y (2014). The effect of licochalcone A on cell-aggregates ECM secretion and osteogenic differentiation during bone formation in metaphyseal defects in ovariectomized rats. Biomaterials..

[34] Flores MG, Hasegawa M, Yamato M, Takagi R, Okano T, Ishikawa I (2008). Cementum-periodontal ligament complex regeneration using the cell sheet technique. J Periodontal Res..

[35] Yang B, Chen G, Li J, Zou Q, Xie D, Chen Y, Wang H, Zheng X, Long J, Tang W, Guo W, Tian W (2012). Tooth root regeneration using dental follicle cell sheets in combination with a dentin matrix-based scaffold. Biomaterials..

[36] Akintoye SO, Lam T, Shi S, Brahim J, Collins MT, Robey PG (2006). Skeletal site-specific characterization of orofacial and iliac crest human bone marrow stromal cells in same individuals. Bone..

[37] Gaur T, Hussain S, Mudhasani R, Parulkar I, Colby JL, Frederick D, Kream BE, van Wijnen AJ, Stein JL, Stein GS, Jones SN, Lian JB (2010). Dicer inactivation in osteoprogenitor cells compromises fetal survival and bone formation, while excision in differentiated osteoblasts increases bone mass in the adult mouse. Dev Biol..

[38] Nelson CM, Bissell MJ (2006). Of extracellular matrix, scaffolds, and signaling: tissue architecture regulates development, homeostasis, and cancer. Annu Rev Cell Dev Biol..

[39] Hannachi IE, Yamato M, Okano T (2009). Cell sheet technology and cell patterning for biofabrication. Biofabrication..

[40] Paschos NK, Brown WE, Eswaramoorthy R, Hu JC, Athanasiou KA (2015). Advances in tissue engineering through stem cell-based co-culture. J Tissue Eng Regen Med..

[41] Szpalski C, Sagebin F, Barbaro M, Warren SM (2013). The influence of environmental factors on bone tissue engineering. J Biomed Mater Res B Appl Biomater..

[42] Chen J, Crawford R, Chen C, Xiao Y (2013). The key regulatory roles of the PI3K/Akt signaling pathway in the functionalities of mesenchymal stem cells and applications in tissue regeneration. Tissue Eng B Rev..

[43] Zhang M, Methot D, Poppa V, Fujio Y, Walsh K, Murry CE (2001). Cardiomyocyte grafting for cardiac repair: graft cell death and anti-death strategies. J Mol Cell Cardiol..

[44] El-Ghalbzouri A, Van Den Bogaerdt AJ, Kempenaar J, Ponec M (2004). Human adipose tissue-derived cells delay re-epithelialization in comparison with skin fibroblasts in organotypic skin culture. Br J Dermatol..

[45] Huh CH, Kim SY, Cho HJ, Kim DS, Lee WH, Kwon SB, Na JI, Park KC (2007). Effects of mesenchymal stem cells in the reconstruction of skin equivalents. J Dermatol Sci..

[46] Michel M, L’Heureux N, Auger FA, Germain L (1997). From newborn to adult: phenotypic and functional properties of skin equivalent and human skin as a function of donor age. J Cell Physiol..

[47] Tan Z, Zhao Q, Gong P, Wu Y, Wei N, Yuan Q, Wang C, Liao D, Tang H (2009). Research on promoting periodontal regeneration with human basic fibroblast growth factor-modified bone marrow mesenchymal stromal cell gene therapy. Cytotherapy..

[48] Xu Q, Li B, Yuan L, Dong Z, Zhang H, Wang H, Sun J, Ge S, Jin Y (2014). Combination of platelet-rich plasma within periodontal ligament stem cell sheets enhances cell differentiation and matrix production. J Tissue Eng Regen Med.

[49] Yu J, Deng Z, Shi J, Zhai H, Nie X, Zhuang H, Li Y, Jin Y (2006). Differentiation of dental pulp stem cells into regular-shaped dentin-pulp complex induced by tooth germ cell conditioned medium. Tissue Eng..

[50] McCulloch CA, Nemeth E, Lowenberg B, Melcher AH (1987). Paravascular cells in endosteal spaces of alveolar bone contribute to periodontal ligament cell populations. Anat Rec..

[51] Wang L, Shen H, Zheng W, Tang L, Yang Z, Gao Y, Yang Q, Wang C, Duan Y, Jin Y (2011). Characterization of stem cells from alveolar periodontal ligament. Tissue Eng A..

[52] Tan SS, Morriss-Kay GM (1986). Analysis of cranial neural crest cell migration and early fates in postimplantation rat chimaeras. J Embryol Exp Morphol..

[53] Serbedzija GN, Bronner-Fraser M, Fraser SE (1992). Vital dye analysis of cranial neural crest cell migration in the mouse embryo. Development..

[54] Schmeichel KL, Bissell MJ (2003). Modeling tissue-specific signaling and organ function in three dimensions. J Cell Sci..

[55] Alvarez-Perez MA, Narayanan S, Zeichner-David M, Rodriguez Carmona B, Arzate H (2006). Molecular cloning, expression and immunolocalization of a novel human cementum-derived protein (CP-23). Bone..

[56] Lai RC, Yeo RW, Lim SK (2015). Mesenchymal stem cell exosomes. Semin Cell Dev Biol..

[57] Rahmi G, Pidial L, Silva AK, Blondiaux E, Meresse B, Gazeau F, Autret G, Balvay D, Cuenod CA, Perretta S, Tavitian B, Wilhelm C, Cellier C, Clement O (2016). Designing 3D mesenchymal stem cell sheets merging magnetic and fluorescent features: when cell sheet technology meets image-guided cell therapy. Theranostics..

