# Correction: Composite cell sheet for periodontal regeneration: crosstalk between different types of MSCs in cell sheet facilitates complex periodontal-like tissue regeneration

**DOI:** 10.1186/s13287-022-03077-7

**Published:** 2022-07-27

**Authors:** Hao Zhang, Shiyu Liu, Bin Zhu, Qiu Xu, Yin Ding, Yan Jin

**Affiliations:** 1 State Key Laboratory of Military Stomatology, National Clinical Research Center for Oral Diseases, Center for Tissue Engineering, School of Stomatology, The Fourth Military Medical University, Xi’an, Shaanxi 710032 People’s Republic of China; 2 State Key Laboratory of Military Stomatology, National Clinical Research Center for Oral Diseases, Shanxxi Clinical Research Center for Oral Diseases, Department of Orthodontics, School of Stomatology, The Fourth Military Medical University, Xi’an, Shaanxi 710032 People’s Republic of China; 3 Research and Development Center for Tissue Engineering, Fourth Military Medical University, Xi’an, Shaanxi 710032 People’s Republic of China; 4 Department of Stomatology, General Hospitalof Tibet Military Region, Lhasa, Tibet 850007 People’s Republic of China; 5 School & Hospital of Stomatology, Wenzhou Medical University, Wenzhou, Zhejiang 325003 People’s Republic of China

## Correction to: Stem Cell Research & Therapy (2016) 7:168 10.1186/s13287-016-0417-x

The original version of the above article contains errors that need to be corrected. Incorrect ALP staining images for Fig. 3a (JBMMSCS group, lower, and CSCS group, upper) were used in figure assembly. The authors now provide the correct images together with the quantification results for ALP staining in Fig. 3a. The corrected Fig. 3, together with the accompanying legend, appears below. Furthermore, the authors have provided a new Supplementary Figure (Additional file 1: Fig. S3, together with the accompanying legend) related to Fig. 3 to show the original gel images of BSP and ALP results in Fig. 3c. The correction does not affect the conclusions of the above paper. We apologize for the mistakes and any inconvenience caused.

**Fig. 3:**
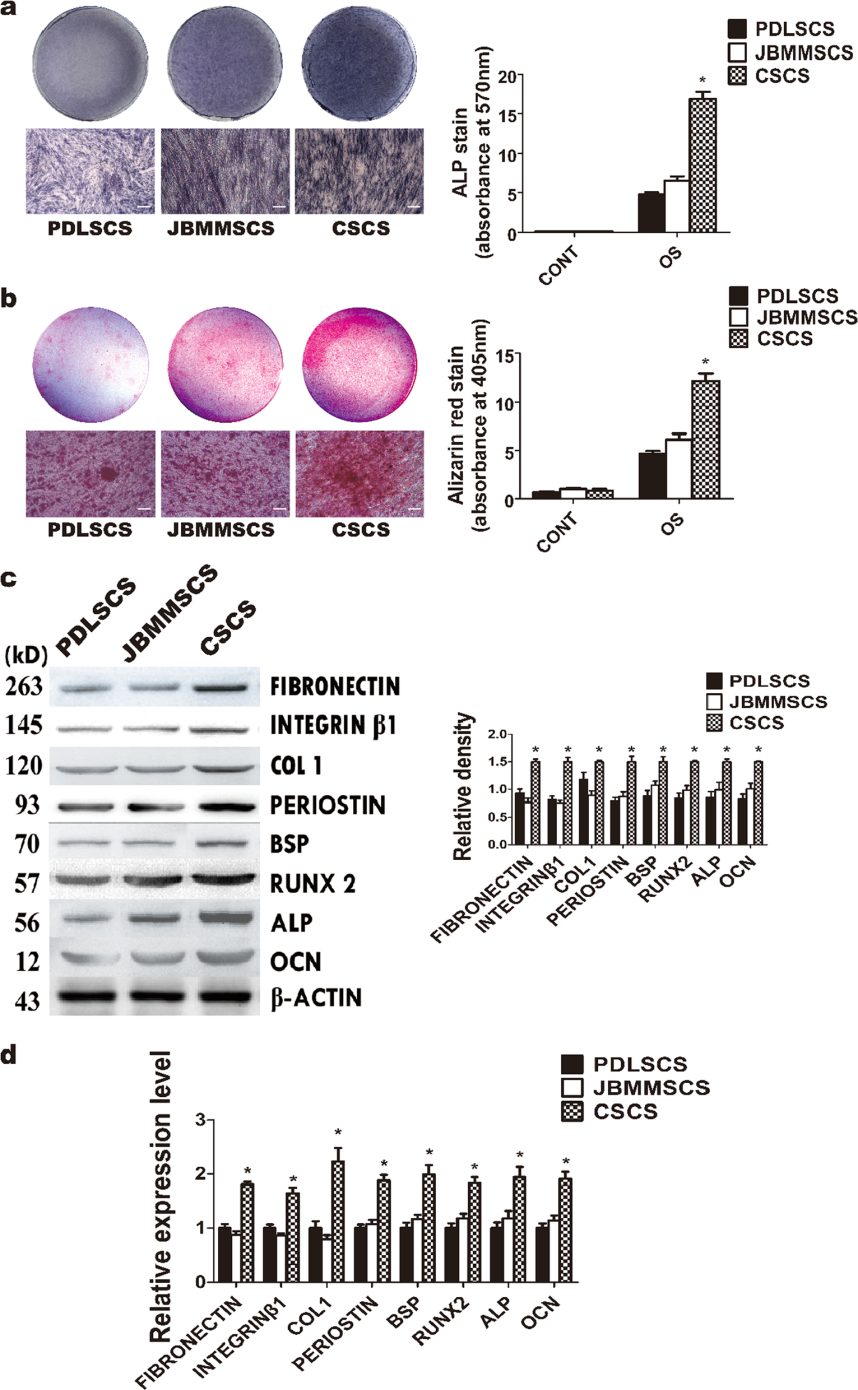
Investigation of the difference of *PDLSCS*, *JBMMSCS* and *CSCS* in vitro. **a** ALP activity of the three types of cell sheets assessed by ALP staining and quantified by absorptiometry. **b** Osteogenic differentiation of the three types of cell sheets assessed by Alizarin red staining and quantified by absorptiometry. **c** The results of Western blot and quantitation show the expression of osteoblast- and ECM-related proteins in the three types of cell sheets. **d** The results of PCR show the expression of osteoblast- and ECM-related genes in the three types of cell sheets. *PDLSCS*: periodontal ligament stem cell sheet; *JBMMSCS*: jaw bone marrow-derived mesenchymal stem cell sheet; *CSCS*: composite stem cell sheet. The data are shown as mean ± SD. * *P* < 0.05, n = 3. The scale bar represents 50 μm

## Supplementary Information

**Additional file 1: Fig. S3**. The original gel images of BSP and ALP results in fig.3c. **a** The original gel image of BSP. **b** The original gel image of BSP with labels and marks. **c** The original gel image of ALP. **d** The original gel image of ALP with labels and marks. *PDLSCS*: periodontal ligament stem cell sheet;* JBMMSCS*: jaw bone marrow-derived mesenchymal stem cell sheet; *CSCS*: composite stem cell sheet; Yellow arrows: the blots displayed in fig.3c (BSP and ALP).

