# Supplementary figures and images for: Composite cell sheet for periodontal regeneration: crosstalk between different types of MSCs in cell sheet facilitates complex periodontal-like tissue regeneration
Source: Stem Cell Res Ther. 2016 Nov 14;7:168. doi: 10.1186/s13287-016-0417-x (PMC5109898; doi:10.1186/s13287-016-0417-x)

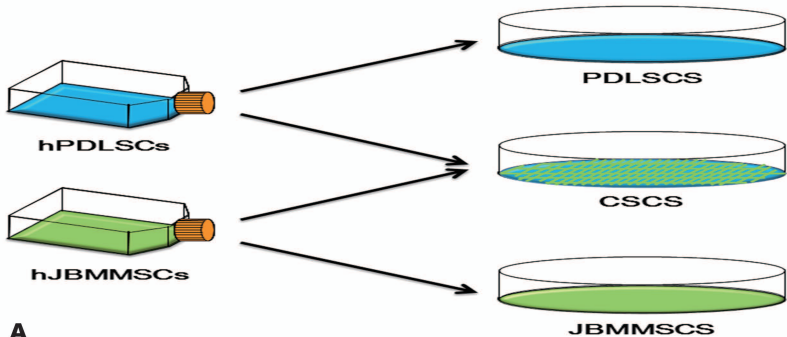

**A**

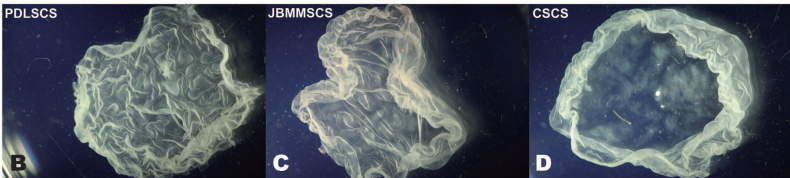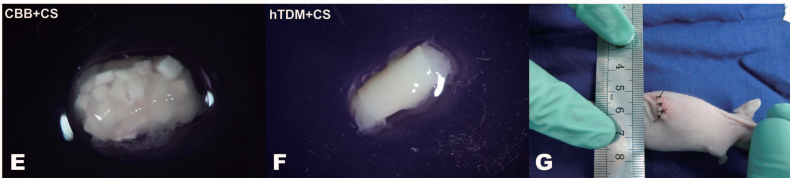

Supplement: Additional file 1: — Construction strategy of different cell sheets and nude mouse ectopic transplantation. (A) Multiple colony-derived hPDLSCs, hJBMMSCs, and the mixed cells of the two above in equal proportions were seeded into six-cell plates, and after induction by conditioned media, (B) PDLSCS, (C) JBMMSCS, and (D) CSCS were formed. (E, F) CBB/hTDM wrapped by cell sheets was cultured as the transplantation grafts. (G) Each mouse received two grafts, one on each side. The wounds were sutured to achieve primary closure. (PDF 596 kb) [file 13287_2016_417_MOESM1_ESM.pdf]
