# Supplementary material for: Composite cell sheet for periodontal regeneration: crosstalk between different types of MSCs in cell sheet facilitates complex periodontal-like tissue regeneration
Source: Stem Cell Res Ther. 2016 Nov 14;7:168. doi: 10.1186/s13287-016-0417-x (PMC5109898; doi:10.1186/s13287-016-0417-x)
Supplement: Additional file 2: — Microscopic appearance of the scaffold materials and the adhesion of the cells to the scaffolds. (A, B) CBB showed a porous structure, and (E, F) the surface of hTDM showed dentinal tubules were sufficiently exposed. (C, D, G, H) The hPDLSCs and hJBMMSCs could adhere to these two scaffolds well, proliferate adequately, and extend excessively on the surface of CBB and hTDM. (PDF 511 kb) [file 13287_2016_417_MOESM2_ESM.pdf]

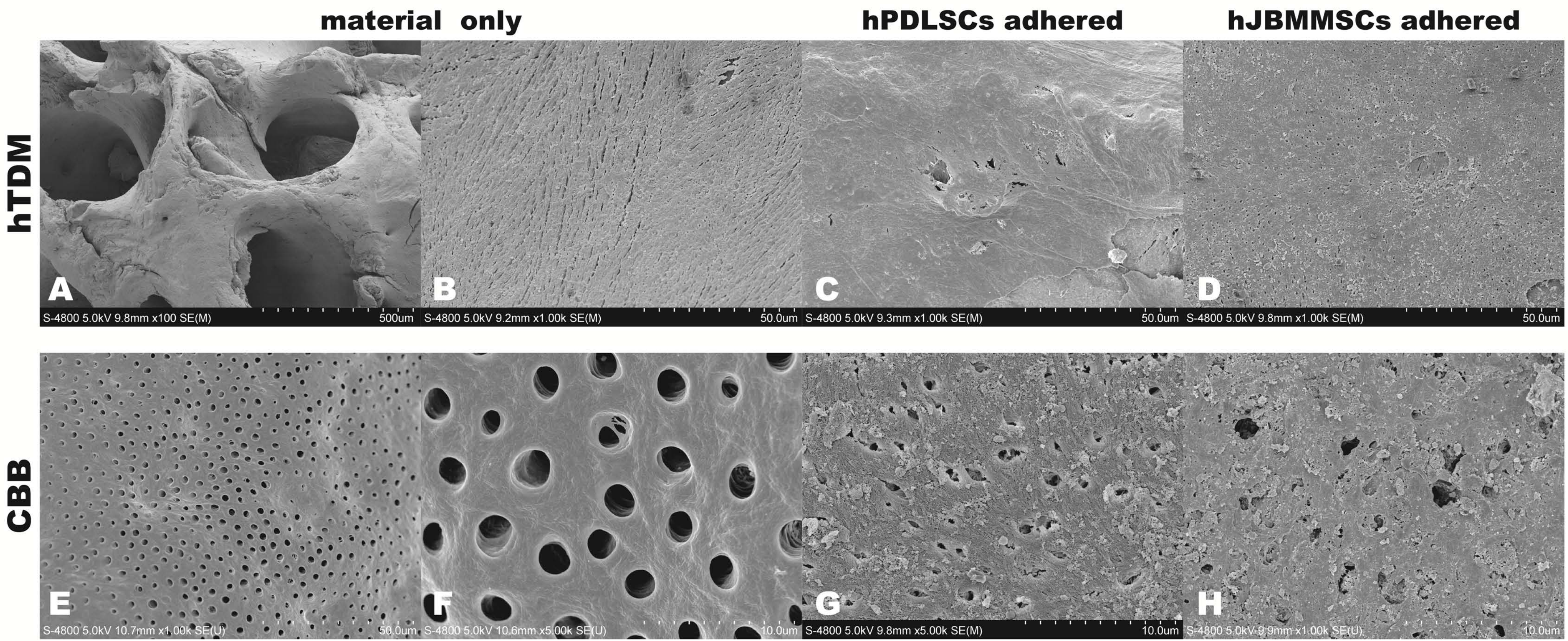
material only
hPDLSCs adhered
hJBMMSCs adhered
hTDM
CBB
A
S-4800 5.0kV 9.8mm x100 SE(M) 500um
B
S-4800 5.0kV 9.2mm x1.00k SE(M) 50.0um
C
S-4800 5.0kV 9.3mm x1.00k SE(M) 50.0um
D
S-4800 5.0kV 9.8mm x1.00k SE(M) 50.0um
E
S-4800 5.0kV 10.7mm x1.00k SE(U) 50.0um
F
S-4800 5.0kV 10.6mm x5.00k SE(U) 10.0um
G
S-4800 5.0kV 9.8mm x5.00k SE(M) 10.0um
H
S-4800 5.0kV 9.9mm x1.00k SE(U) 10.0um
