# Supplementary material for: Correction: Composite cell sheet for periodontal regeneration: crosstalk between different types of MSCs in cell sheet facilitates complex periodontal-like tissue regeneration
Source: Stem Cell Res Ther. 2022 Jul 27;13:363. doi: 10.1186/s13287-022-03077-7 (PMC9327228; doi:10.1186/s13287-022-03077-7)
Supplement: Supplementary file 1 — Additional file 1: Fig. S3. The original gel images of BSP and ALP results in fig.3c. a The original gel image of BSP. b The original gel image of BSP with labels and marks. c The original gel image of ALP. d The original gel image of ALP with labels and marks. PDLSCS: periodontal ligament stem cell sheet; JBMMSCS: jaw bone marrow-derived mesenchymal stem cell sheet; CSCS: composite stem cell sheet; Yellow arrows: the blots displayed in fig.3c (BSP and ALP). [file 13287_2022_3077_MOESM1_ESM.docx]

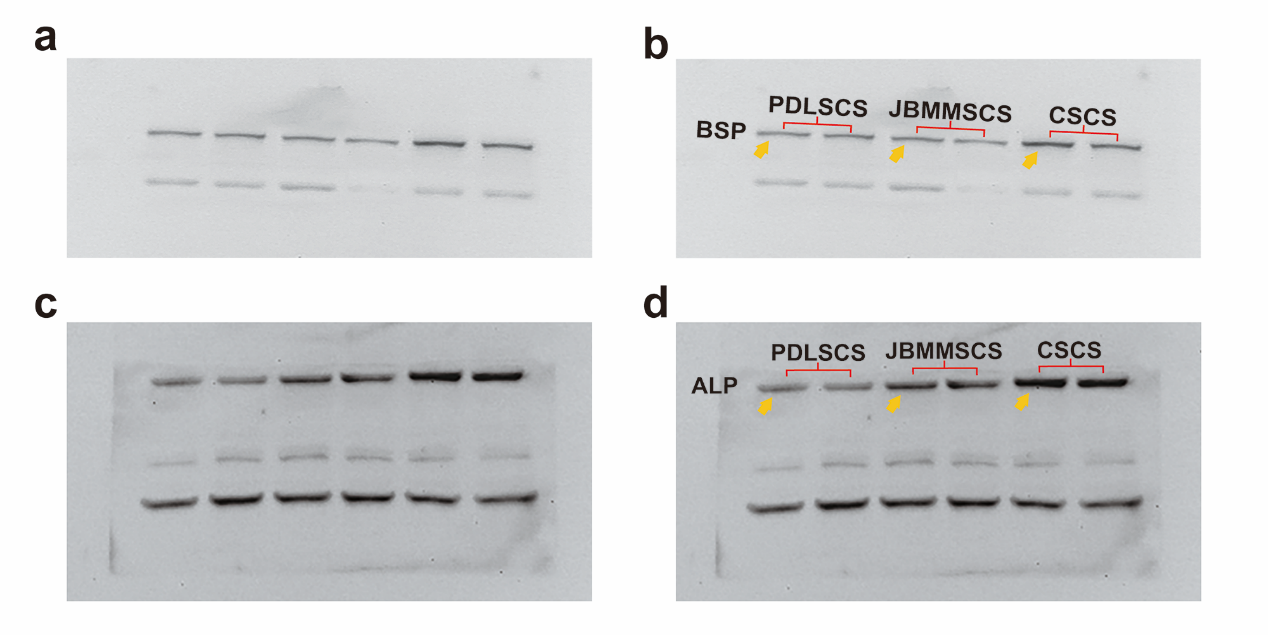


**Supplementary Figure 3.** The original gel images of BSP and ALP results in fig.3c. **a** The original gel image of BSP. **b** The original gel image of BSP with labels and marks. **c** The original gel image of ALP. **d** The original gel image of ALP with labels and marks. *PDLSCS*: periodontal ligament stem cell sheet; *JBMMSCS*: jaw bone marrow-derived mesenchymal stem cell sheet; *CSCS*: composite stem cell sheet; Yellow arrows: the blots displayed in fig.3c (BSP and ALP).
